# How does collectivism help deal with perceived vaccine artificiality? The case of COVID-19 vaccination intent in European young adults

**DOI:** 10.1371/journal.pone.0300814

**Published:** 2024-03-19

**Authors:** Wojciech Trzebiński, Jerzy Trzebiński

**Affiliations:** 1 SGH Warsaw School of Economics, Warsaw, Poland; 2 SWPS University, Warsaw, Poland; BRAC Business School, BRAC University, BANGLADESH

## Abstract

Vaccine "unnaturalness" (artificiality) is one of the major anti-vaccine arguments raised in public debate. Therefore, health communication should rebut unnaturalness arguments and be cautious when emphasizing human intervention (e.g., advanced vaccine technology), which may entail perceiving vaccines as artificial. Understanding how the relationship between perceived vaccine artificiality and vaccination intent differs across social groups can help enforce the above health communication efforts by focusing them on specific audiences. The objective of the current paper is to assess the moderating role of a particular socio-cultural factor—vertical collectivism (reflecting the orientation on social hierarchy)—in the relationship between perceived vaccine artificiality and vaccination intent. It is proposed that vertical collectivism diminishes the negative effect of perceived vaccine artificiality. Two studies with European young adults measured COVID-19 vaccination intent and vertical collectivism. Study 1 (N = 418) was correlational, measuring perceived vaccine artificiality. The data were analyzed with a moderation model. Study 2 (N = 203) was experimental, manipulating perceived vaccine artificiality by human-intervention appeal (i.e., emphasizing human intervention in vaccine development and operation). The data were analyzed with moderation and moderated mediation models. Study 1 demonstrated that the effect of perceived vaccine artificiality on vaccination intent was less negative when the level of vertical collectivism was higher. In Study 2, with higher levels of vertical collectivism, the effect of human-intervention appeal on vaccination intent was less negative, and the indirect effect through perceived vaccine artificiality turned even positive. Those results contribute to the fields of perceived naturalness/artificiality, vaccination behavior, health communication, and cultural dimensions theory, providing empirical evidence that the negative effect of perceived vaccine artificiality on vaccination intent is diminished by vertical collectivism, as proposed. Health practitioners are guided on how to consider different levels of collectivism of their audiences while referring to vaccine artificiality in their communication. Specifically, it is suggested that rebutting "unnaturalness" anti-vaccine arguments should be focused on people low in vertical collectivism, and messages featuring human intervention (e.g., a vaccine’s technological advancement) should be targeted at people high in vertical collectivism.

## Introduction

Vaccine hesitancy has long been posing a key challenge for vaccination programs [1], and one of the factors lowering vaccination intent is a general perception of vaccines as unnatural (artificial) [2, 3]. Namely, the idea of vaccines is that they intervene in the body before the infection [3], which may appear as going against the "natural" processes in which illnesses spread and are "naturally" fought by human bodies. Given that perception, it is crucial for health communication to understand why people tend to react negatively to perceived vaccine "unnaturalness" (artificiality). To the best of our knowledge, the existing literature has not sufficiently addressed this issue, as little is known about the underlying determinants of people’s response to perceived vaccine artificiality. Therefore, we offer a novel mechanism based on a socio-cultural construct, vertical collectivism [4–12], defined as the degree to which people acknowledge their dependency within the social hierarchy. In this article, we propose and empirically examine that vertical collectivism makes people react to perceived vaccine artificiality less negatively. From the practical perspective of health communication, it suggests that efforts to rebut anti-vaxxers’ "unnaturalness" arguments should focus on people low in vertical collectivism while emphasizing human achievements in intervening in nature should focus on people high in vertical collectivism. Below, we present our idea in more detail.

A wide range of vaccination intent determinants has been heavily studied, including perceptual constructs like perceived vaccine unnaturalness (artificiality) [2, 3] and socio-cultural ones, like collectivism [4–12]. While each of those two constructs was, separately, demonstrated to be an important antecedent of vaccination intent, their interplay remains unclear. However, if one views vaccines as a means of human (societal) activity to fight diseases, the above antecedents, i.e., perceived vaccine artificiality and collectivism, have an important thing in common: they both can be related to the human (societal) activity in developing vaccines. Namely, perceived vaccine artificiality pertains to the degree to which a vaccine is considered as a result of such activity, and collectivism refers, in general, to acknowledging one’s dependency on the activities of other people in society. This connection between perceived vaccine artificiality and collectivism raises a question about the possibility that those two variables work together (interplay) in shaping vaccination intent. Therefore, this paper aims to investigate that possibility by theoretically deriving such interplay, examining it empirically in two studies, and discerning its practical meaning.

While collectivism has been consistently found to increase vaccination intent [4–12], the perception of vaccine unnaturalness (artificiality) constitutes a challenge for vaccination programs. That is, previous studies showed vaccines to be perceived mainly as unnatural (artificial) [3], which contributed to vaccination hesitancy [2]. Moreover, vaccine "unnaturalness" was identified as one of the main anti-vaxxers’ arguments [13]. It is, therefore, paramount to identify ways to diminish the negative effects of perceived vaccine artificiality. Specifically, vaccine communication needs to rebut "unnaturalness" arguments (e.g., by stating that vaccination is based on natural processes) and be cautious in using human-intervention appeals (i.e., the messages emphasizing human intervention behind vaccine development and operation, e.g., featuring advanced technology [14, 15] of a vaccine), which may entail perceiving vaccines as more artificial. It is crucial for vaccine promoters to know on whom they should focus their efforts to rebut the "unnaturalness" arguments and on whom they should focus vaccine human-intervention appeals. Understanding the above is especially important given that those efforts may be costly (e.g., when they are based on personal communication or social media campaigns) or counterproductive (as in the case of human-intervention appeals). Particularly, identifying socio-cultural moderators of the effect of perceived vaccine artificiality on vaccination intent can help vaccine promoters focus their efforts on certain groups of health communication recipients. However, the existing literature on vaccination intent provides little guidance on that, and particularly, it does not sufficiently explain whether collectivism can play any role in shaping the effect of perceived vaccine artificiality on vaccination intent. Meanwhile, cultural dimensions theory [16] emphasizes the function of cultural collectivism in defining interdependencies within the society, among others, between groups of different levels in the social hierarchy. We apply those theoretical considerations to the vaccine context, where high-level social groups are responsible for developing and deploying "artificial" vaccines to fight "natural" epidemics. We propose that the degree to which people perceive and acknowledge social hierarchy (which is represented by a subdimension of collectivism called vertical collectivism) reduces the negative effects of perceiving vaccine artificiality on vaccination intent. We, therefore, consider vertical collectivism as a potential remedy to the challenge of the general perception of vaccines as "unnatural" (artificial). Specifically, in two studies, we test whether vertical collectivism diminishes the negative effect of perceived vaccine artificiality. Study 1 was correlational and focused on the perceived artificiality of vaccines (in general) as a personal characteristic. In contrast, Study 2 experimentally manipulated the artificiality perception of a particular vaccine by a human-intervention appeal (i.e., emphasizing human intervention behind vaccine production and operation). Both studies measured the individual level of vertical collectivism and demonstrated that the effect of vaccine artificiality perception on vaccination intent was less negative (or even more positive) when the level of vertical collectivism was higher. Those results contribute to the fields of perceived naturalness/artificiality, vaccination behavior, health communication, and cultural dimensions theory by providing empirical evidence that the negative effect of perceived vaccine artificiality on vaccination intent is diminished by vertical collectivism. Our research informs practitioners responsible for vaccine communication on audiences who are likely to be more prone to anti-vaxxers’ "unnaturalness" arguments and not respond positively to emphasizing human intervention in vaccine communication (i.e., people with low vertical collectivism), as well as audiences who are likely to be resilient to unnaturalness arguments and respond positively to human-intervention appeals (i.e., people high in vertical collectivism).

The remainder of the paper starts with reviewing the determinants of vaccination intent and the concept of unnaturalness (artificiality) as applied to vaccines. Next, we present the theoretical underpinning of our considerations (i.e., cultural dimensions theory) and explain our focus on vertical collectivism as a potential moderator of the effect of perceived vaccine artificiality on vaccination intent. We then report our studies aiming to test the moderating role of vertical collectivism, where vaccination intent was a dependent variable, and the individual level of perceived vaccine artificiality (Study 1) or the human-intervention appeal in vaccine communication (Study 2) was an independent variable. The paper ends with formulating the theoretical and practical implications, limitations, and directions for further research.

## Theoretical background

### Determinants of vaccination intent

Even though vaccines may effectively respond to epidemics, vaccine hesitancy remains a considerable phenomenon [17–20]. Taking the European example, nearly one year after COVID-19 vaccines became available (i.e., at the end of 2021), and amongst over six million total death toll of SARS-CoV-2 in the European Economic Area (EEA) [17], less than 69% of EU citizens were vaccinated against COVID-19 (primary course), with even lower levels in some countries like Bulgaria (28%) [18]. According to Eurobarometer data for 2022 [19], 23% of EU citizens disagreed, tended to disagree, or were unsure whether COVID-19 benefits outweigh the risks. Such considerable uncertainty pertains not only to vaccines against COVID-19 but to vaccines in general. For example, the percentage of citizens considering vaccines safe in Bulgaria was only 66% [20].

The existing literature has heavily studied different kinds of vaccination intent determinants. A large bunch of research on this topic deals with vaccine-related attitudinal factors, like trust in vaccines, perceived risks associated with preventable illnesses, perceived benefits and costs of vaccination [21–25], emotions [26], and perceived vaccine artificiality [2, 3]. Another research stream focuses on health communication, specifically social and media communication [24, 25, 27–29], advertising [30, 31], risk disclosure [32], and message design [33, 34]. Studies also investigate the role of individual characteristics, including demographics [35], previous vaccination history [36], and socio-cultural determinants, like political conservatism [37–39], sophistication [38], beliefs about the world’s orderliness and positivity [40, 41], and collectivism [4–12].

In sum, previous studies considered three categories of vaccination intent determinants: vaccine-related attitudinal factors, health communication, and individual characteristics, including socio-cultural determinants. In the current research, those categories are represented by perceived vaccine artificiality, human-intervention appeal (as a basis of message design), and collectivism, respectively.

### Perceived vaccine artificiality

While the concept of the perceived unnaturalness (artificiality) of an entity may seem intuitive, it is not easy to establish a precise formal definition [42] because many aspects can be referred to as "natural" vs. "unnatural" or "artificial." One aspect is the entity’s properties; property-based perceived (un)naturalness is the degree to which a person considers certain features of an entity as "(un)natural" [42, 43], e.g., (un)related to natural (biological) life (cf. [3]). Another aspect of the entity’s (un)naturalness is related to its history [42, 43], specifically, the degree to which a person considers the origination of the entity to be based on (free of) human involvement. Accordingly, in this paper, we define perceived vaccine unnaturalness (artificiality) as the degree to which a person considers a vaccine as originated or operating based on a human-planned intervention (e.g., using advanced technology) vs. being based on natural (biological) components and natural (biological) processes through which the vaccine operates within the organism.

People generally prefer more "natural" products and brands [44], including more "natural" drugs (e.g., [45, 46]). Perceived naturalness was identified as an important food choice determinant [47] and the reason for the preference for herbal medicines and beverages [48]. This effect is referred to as naturalness bias, which may result from people’s belief that naturalness is a positive property (a "natural-is-better" default belief) and is instrumental to specific goals like safety [49]. For example, people may consider naturalness as a low-risk cue, associating higher risks with unnatural objects. Li & Chapman [46] demonstrated that people perceive a higher risk of undesired health consequences of unnatural (vs. natural) allergens and a medicine (vitamin C).

Naturalness bias was also demonstrated for vaccines. People may perceive vaccines as more or less unnatural [50], and vaccine unnaturalness perception is negatively related to vaccination attitudes [2]. Likewise, one of the main concerns raised by anti-vaxxers is the issue of vaccine "unnaturalness," including the perception of vaccines as based on human intervention in the natural world [3, 13]. People were less willing to take a COVID-19 vaccine when they had a higher preference for "natural" food [51] and medicines [49] (i.e., a higher naturalness bias) and when they perceived vaccines as more unnatural, which characterized vaccine rejecters [2]. Those results suggest that vaccines are generally perceived as artificial, this perception contributes to vaccine hesitancy, and that the level of perceived vaccine artificiality and the strength of naturalness bias vary among people.

Although previous research provides some insights on how individual characteristics may shape vaccine naturalness bias, e.g., declared preference for natural immunity was positively related to education [52], the moderators of the relationship between perceived vaccine naturalness and vaccination intent remain understudied and, to the best of our knowledge, no research studied socio-cultural moderators. This gap is especially important as it limits our understanding of how the consequences of vaccine-related public discourses (e.g., "unnaturalness" anti-vaxxers’ arguments) and communication efforts (e.g., vaccine promotional messages featuring human intervention) may differ across audiences. To bridge this gap, we focus on a particular socio-cultural characteristic ‒ vertical collectivism [53] as a potential moderator of the perceived artificiality’s effect on vaccination intent. In the next section, we present the concept of vertical collectivism and explain why it could be particularly useful to identify the variability of the vaccine artificiality perception effects.

### Vertical collectivism as a socio-cultural moderator of the effect of perceived vaccine artificiality

Cultural dimensions theory [16] posits that culture, as a complex set of "values, beliefs, norms, and self-descriptions" (p. 11), is structured in the form of several independent dimensions that were originally formulated as collectivism vs. individualism (related to equal and hierarchical relationships within the group), power distance (related to the relationship with authority), masculinity-femininity (related to social implications of gender), and uncertainty avoidance (related to ambiguous and unknown situations). Later research successfully applied those dimensions at the individual level [53, 54], demonstrating its role, e.g., in people’s response to product communication. Drawing on that theoretical foundation, we elaborate below on how the effect of perceiving vaccines as artificial may be socio-culturally moderated. Our initial assumptions were that (1) developing vaccines is a societal effort to fight epidemics, led by social groups high in the social hierarchy, like scientists and governments; (2) vaccine artificiality reflects the degree of this societal effort, i.e., human intervention in the natural disease. Cultural orientation to the social hierarchy (related to collectivism vs. individualism and power distance) should be, therefore, relevant to investigate the consequences of vaccine artificiality perception.

Collectivism (vs. individualism) is defined as the degree to which people perceive themselves based on their relationships with other people and social roles [53, 55]. Collectivist people tend to value society’s well-being over the individual’s [56] and emphasize a society based on "collective satisfaction," "collective outcomes," and shared interests [57]. Collectivism is related to prosocial orientation and caring for others [53]. Accordingly, numerous studies demonstrated the positive relationship between collectivism and health-prevention behavioral tendencies [58], including vaccination intent [4–12]. Collectivism was also found to play a moderating role in shaping vaccination intent. Specifically, collectivism may strengthen the link between vaccine intent and vaccine attitudes [59] and subjective norms [60, 61] and diminish the effect of perceived invincibility on vaccination intent [62]. However, to the best of our knowledge, no previous study assessed how collectivism may modify the consequences of perceived vaccine artificiality.

**Fig 1:**
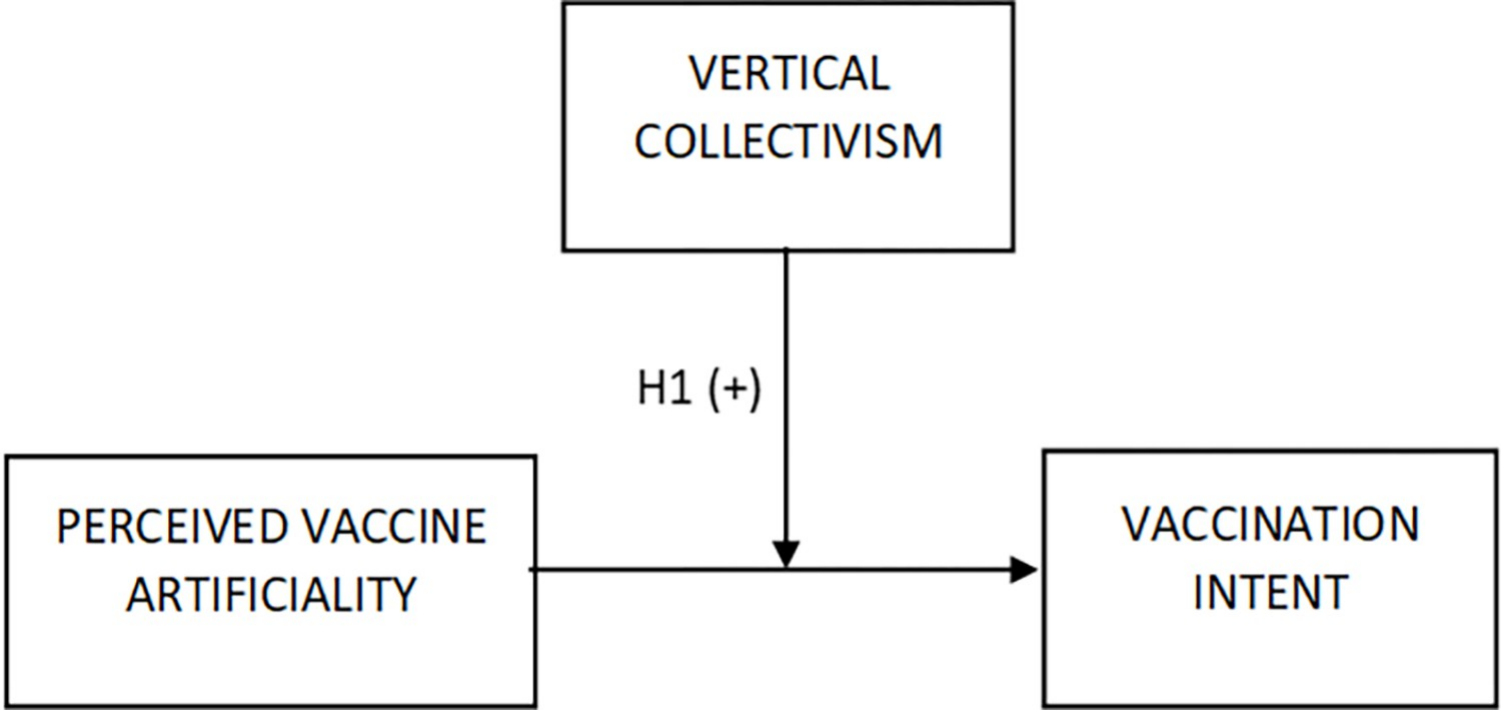
Conceptual model.

Vertical collectivism is a subdimension of collectivism reflecting the orientation on social hierarchy [53]. People high in vertical collectivism acknowledge different levels and roles within society [63]. When applied to the vaccination context, vertical collectivism may lead to higher recognition, appreciation, and acceptance of high-status social roles of people who know "more and better," such as medical scientists, physicians, pharmacists, health policy experts, and policymakers. Consequently, one may propose that people higher in vertical collectivism are more likely to recognize and appreciate inventing, producing, and distributing vaccines as those activities may be perceived as endeavors of those high-level social groups and organizations such as governments, academia, pharmaceutical companies, and healthcare institutions [64–66]. In other words, vertical collectivists may recognize the position of those groups to lead society’s actions against epidemics. Accordingly, trust in social institutions like government, science, and medicine is found to be positively related to vaccine attitudes [67, 68]. Therefore, vertical collectivistic people may wish to use vaccines as a form of support that those high-level social groups provide to overcome the "natural" threat of a contagious illness. Consequently, in that vertical-collectivist perspective, the vaccine "unnaturalness" (artificiality) issue may become less critical as it is resolved by recognizing society as eligible to make preventive efforts during epidemics. Moreover, vertical collectivism may even make people value vaccine "artificiality," as it is based on the activities of the abovementioned high-level social groups, e.g., in technology advancement. This way, for vertical collectivists, vaccine "artificiality" may express highly appreciated societal efforts to fight "natural" diseases and epidemics. In sum, vertical collectivism may reduce naturalness bias, i.e., the negative effect of perceived vaccine artificiality on vaccination intent. Accordingly, it is hypothesized that (Fig 1):

**H1.** The relationship between perceived vaccine artificiality and vaccination intent is less negative for higher levels of vertical collectivism.

As mentioned above, one more of Hofstede’s cultural dimensions, i.e., power distance, is directly related to social hierarchy. Specifically, power distance is defined as accepting unequally distributed power in society [53, 69]. To our theorization presented above, however, vertical collectivism should be more relevant, as it is more generally related to respecting high-status social groups [63]. Namely, the mechanism we propose is not based on obeying mandatory vaccinations (as a manifestation of power) but on recognizing and appreciating the activity of high-status social groups standing behind vaccination programs.

### Overview of the studies

Our hypothesis was tested in two studies, a correlational one (Study 1) and an experiment (Study 2). In the next sections, we present each study separately, including its method (participants, procedure, and measurements), results, and discussion. Here, we provide the general idea and justification for the studies.

Both studies aimed to investigate the predicted positive interaction effect of vertical collectivism and perceived vaccine artificiality on vaccination intent. In other words, the studies had to examine whether vertical collectivism indeed shaped the effect of perceived vaccine artificiality, making the latter less harmful to people’s intentions regarding vaccination. However, Studies 1 and 2 approached this aim from different angles. Namely, Study 1 considered perceived vaccine artificiality as a personal characteristic in the form of a perception that vaccines (in general) are "artificial," assuming that people may differ in that belief, which may result, among others, from different exposure to anti-vaxxers "unnaturalness" arguments. On the other hand, Study 2 focused on a specific type of vaccine communication promoting a particular vaccine by emphasizing human achievements in intervening in nature with that vaccine (human-intervention appeal), which may raise the perceived artificiality of that vaccine. Likewise, brands were perceived as less "natural" when presented through socially-related (vs. environmentally-related) messages [70]. In the context of technology-based products (like vaccines), marketers can use scientifically sound messages [71–73], emphasizing product technological advancement [14, 15], which may suggest the products’ "unnaturalness" (artificiality) [50, 74]. In general, the artificiality may be suggested by stressing human intervention [42, 43]. Accordingly, Study 2 manipulated perceived vaccine artificiality by including (vs. not including) human-intervention appeal.

In both cases ‒ i.e., the perceived artificiality of vaccines in general, considered a personal characteristic (Study 1) and the perceived artificiality of a specific vaccine, resulting from human-intervention appeal (Study 2) ‒ we expected vertical collectivism to diminish the negative consequences of perceived vaccine artificiality on vaccination intent.

## Study 1

### Method

Study 1 aimed to test H1 by investigating the relationship between perceived vaccine artificiality, vaccination intent, and vertical collectivism based on correlational data. As the perception of vaccines as being artificial may vary along with ’ ’people’s different experiences and dispositions, Study 1 used the measured perceived vaccine artificiality as an independent variable. Study 1 obtained the required approval from the institutional review board (SGH Warsaw School of Economics Ethical Committee, approval no. 2/2022).

#### Participants

Four hundred eighteen European young adults aged between 18 and 35, working or studying, with at least high school education, and good self-reported English level (M_age_ = 22.239, SD = 2.796, 53.8% females) participated in an online survey (see sample characteristics in S1 Table). The participants were recruited by a team of marketing research students, like Glaser & Reisinger [75], and written informed consent was obtained via the online survey form. The researchers had no access to information that could identify individual participants during or after data collection. The sample was convenient and homogenous as the study focused on investigating relationships between constructs [76]. Data were collected in October 2021, when 65.7% of the European Union (EU)/European Economic Area (EEA) adult population was vaccinated against COVID-19 with two doses [18]. The data collection was part of a larger project on vaccination intent determinants; results related to other factors (i.e., perceived vaccine novelty and beliefs about the ’ ’world’s positivity and orderliness) were reported by Trzebiński & Trzebiński [41]. The data were accessed for research purposes in November 2021, after the data collection had been completed.

#### Procedure

At the beginning of the questionnaire, the instruction referred to COVID-19 vaccinations in general. To engage the participants, the instruction stated that one might still need to decide whether to vaccinate against COVID-19 even if one had already been vaccinated. The measurements started with a dependent variable to reduce self-generated validity issues [77, 78]. First, the participants rated their intent to vaccinate against COVID-19, then the artificiality of COVID-19 vaccines. Next, the participants reported their level of collectivism and their analytical vs. intuitive cognitive style that reflects their individual disposition in information processing [79]. Analytical thinking style was controlled as it is considered to be related to collectivism (e.g., [53, 80, 81]) and vaccination intent (e.g., [82, 83]). The questionnaire ended with demographics.

#### Measurements

Vaccination intent was measured with two seven-point items adapted from Hopfer [84] (coded from 1 = "definitely NO" to 7 = "definitely YES"), "Supposing you are advised to get the COVID-19 vaccine …I intend to get vaccinated for COVID-19 … if the COVID-19 vaccine is free, I like to get the vaccine" (r = .842, p < .001; ρ = .819, *p* < .001).

Perceived vaccine artificiality was measured with two seven-point items (reverse-coded from 1 = "definitely YES" to 7 = "definitely NO") related to the vaccine properties, like natural components and operating in a natural way. The first item, "Although human-created, COVID-19 vaccines immunize people in a rather natural way"," was adapted from Tenbült et al. [85]. The second item, "COVID-19 vaccines use natural components and mechanisms"," was developed for this study (r = .514, p < .001; ρ = .520, *p* < .001). As the responses were reverse-coded, higher measurement values indicated higher levels of perceived vaccine artificiality.

Collectivism was measured with sixteen seven-point items adapted from Singelis et al. [63] (coded from 1 = "definitely NO" to 7 = "definitely YES"). This measurement included two subscales. The first one measured vertical collectivism as a focal construct involved in our conceptual model (eight items, α = .816; exemplary item: "I would sacrifice an activity that I enjoy very much if my family did not approve it."). To assess the discriminant validity across the collectivism subdimensions, we also measured horizontal collectivism using the second subscale (eight items, α = .906; exemplary item: "The well-being of other people I work with is important to me."). Exploratory Factor Analysis (EFA) with all the sixteen items (factors extracted using Principal Component Analysis (PCA), Kaiser-Meyer-Olkin (KMO) test for sampling adequacy = .907, ’Bartlett’s *p* < .001) revealed two factors with eigenvalues above 1. The items loaded on the factors (loadings above .5, Varimax rotation) in accordance with the subscales, except for one item from the vertical collectivism subscale, "Children should feel honored if their parents receive a distinguished award." This item was removed from the further analysis.

The analytical cognitive style was measured with four seven-point items adapted from Gaston-Breton & Duque [86] (α = .837). The respondents were asked to describe how they typically made decisions when they "thought about doing things" (exemplary item: "I carefully compare the options I have on several different aspects."). The responses were coded from 1 = "definitely NO" to 7 = "definitely YES."

All the measurement scales (vaccination intent, perceived vaccine artificiality, vertical collectivism without the dropped item, and analytical thinking style) were subjected to Confirmatory Factor Analysis (CFA). After dropping three vertical-collectivism items "("Before taking a major trip, I consult with most members of my family and many friends.", "Children should be taught to place duty before pleasure.", "I hate to disagree with others in my group."), the model showed acceptable fit characteristics (χ^2^(48) = 77.397, p < .001, χ^2^/df = 1.612, Root Mean Square Error of Approximation (RMSEA) = .038, Comparative Fit Index (CFI) = 0.985, Tucker Lewis Index (TLI) = 0.980, Standardized Root Mean Square Residual (SRMR) = 0.041). In support of the reliability and convergence validity of the measurement scales, Composite Reliabilities (CRs) and Average Variances Extracted (AVEs) were satisfactory for all scales (CRs ranged from .7 to .9, AVEs ranged from .5 to .8). According to Fornell-Larcker’s criterion, the absolute values of the correlation coefficients between latent variables were lower than the corresponding AVE square roots, and heterotrait-monotrait (HTMT) ratios were below .6 (i.e., within the acceptable threshold of .9), supporting discriminant validity [87]. The factor with the highest eigenvalue in Exploratory Factor Analysis based on the reduced set of indicators (factors extracted using principal component analysis (PCA), Kaiser-Meyer-Olkin (KMO) test for sampling adequacy = .752, Bartlett’s p < .001) explained 29.7% of the variance, suggesting no issues with common method bias [88]. The reduced vertical-collectivism scale remained reliable, α = .782 (see details in S2–S4 Tables). For further analysis, each measurement scale was pooled into a single index.

### Results

**Fig 2:**
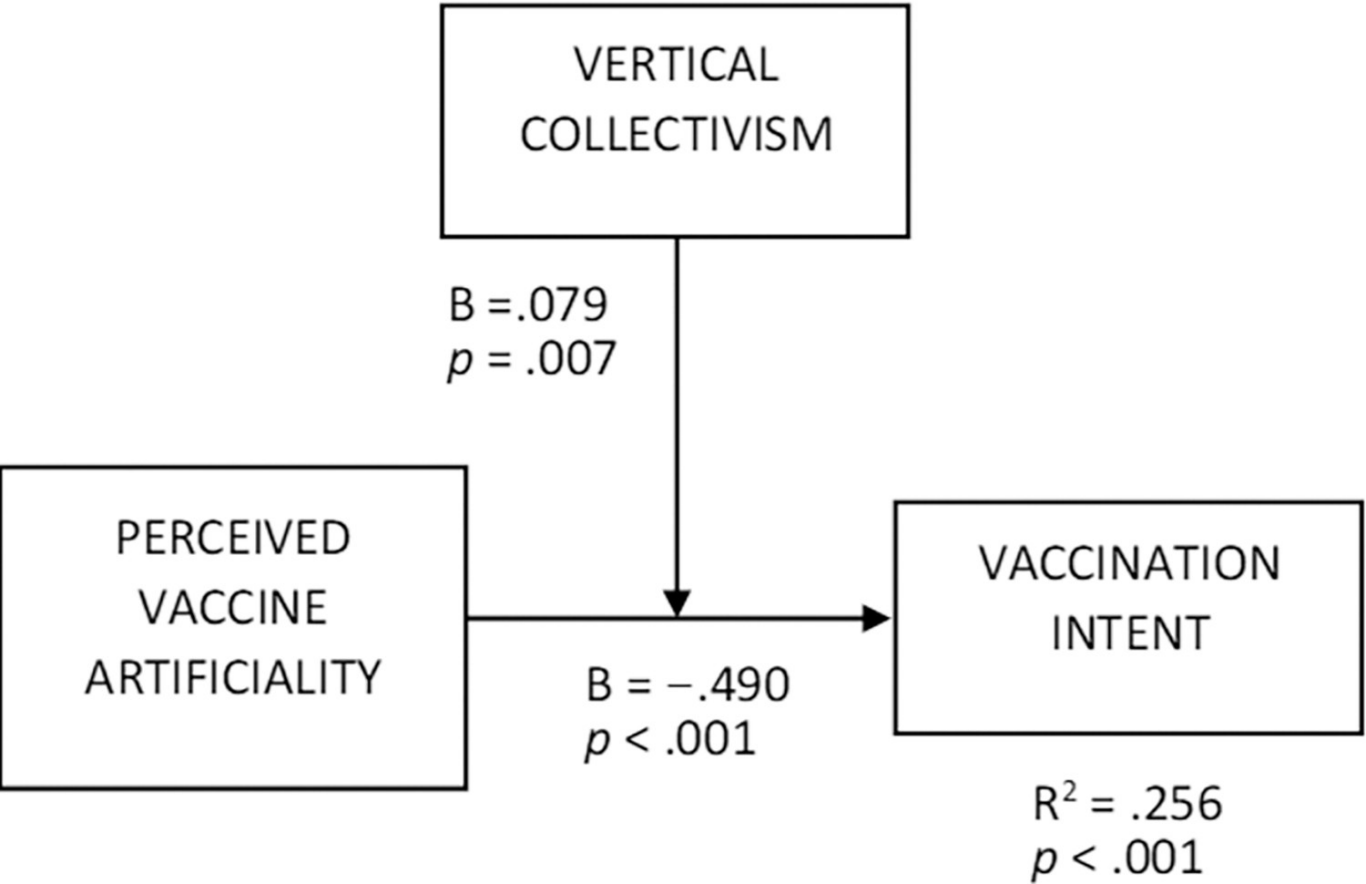
The moderation effect of vertical collectivism on the relationship between perceived vaccine artificiality and vaccination intent (Study 1).

**Fig 3:**
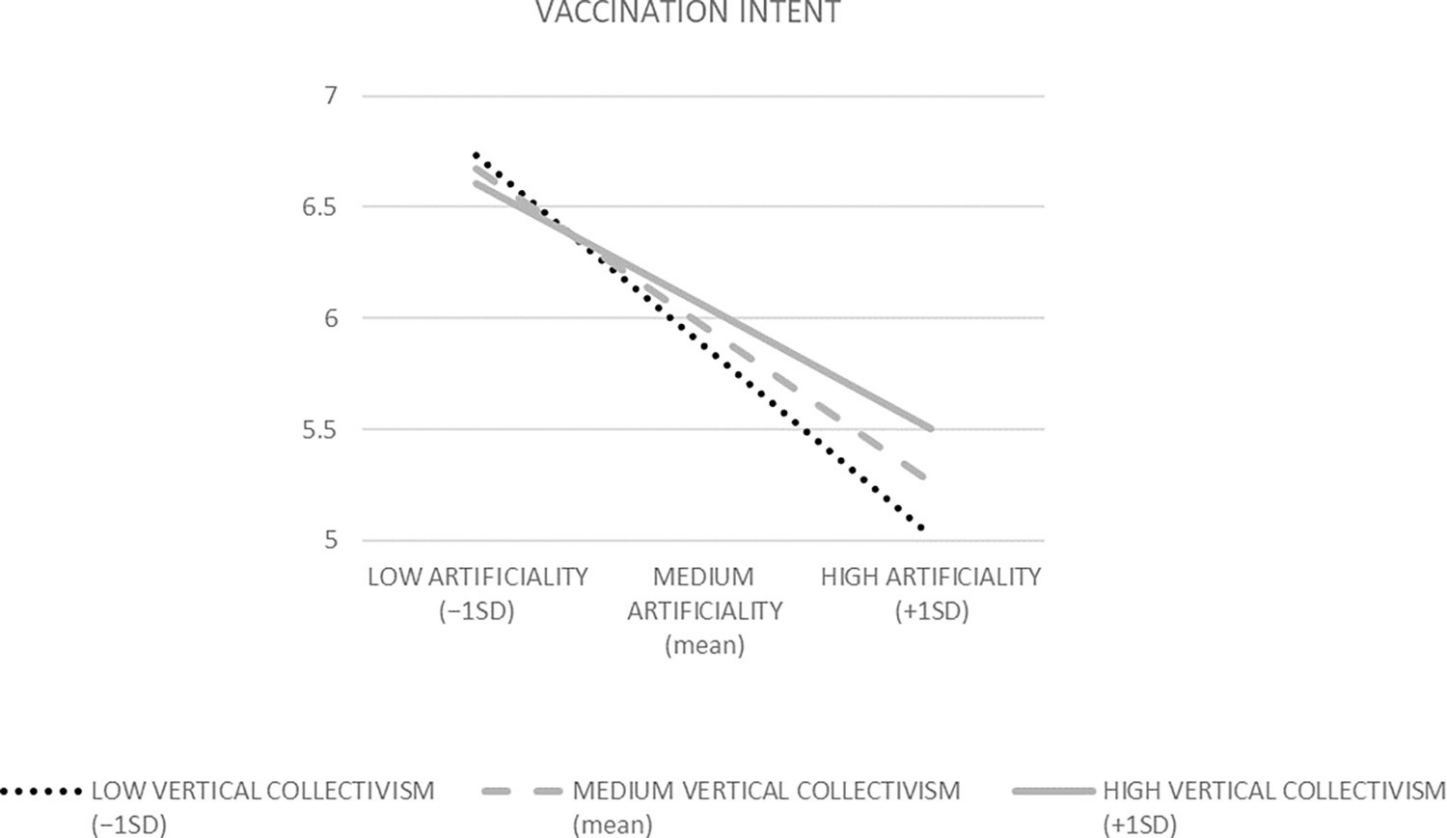
The visualization of conditional effects of perceived vaccine artificiality on vaccination intent; vertical collectivism is the moderator (Study 1).

We applied moderation analysis to test H1, which predicted the positive interaction effect of vertical collectivism and perceived vaccine artificiality on vaccination intent. Specifically, we checked whether the effect of the perceived artificiality of vaccines in general on vaccination intent is less negative for higher levels of vertical collectivism. To this end, we used Ordinary Least Squares (OLS) regressions in PROCESS macro [89], which are suitable for analyzing interactions between continuous variables [90]. In the PROCESS model 1, perceived vaccine artificiality was an independent variable, vertical collectivism was a moderator, and vaccination intent served as a dependent variable, while analytical thinking style, gender (coded as 1 = female, 0 = male), and age were covariates (Fig 2, R^2^ = .256, F = 23.580, *p* < 0.001, Variance Inflation Factors (VIFs) < 1.1). The perceived vaccine artificiality × vertical collectivism interaction effect was positive (B = .079, t = 2.713, *p* = .007), as expected. The conditional effects of perceived vaccine artificiality increased with the higher values of vertical collectivism. Namely, at the vertical collectivism level of −1SD, the effect was negative (B = -.596, t = 9.350, *p* < .001). At the mean level of vertical collectivism level, the effect was less negative (B = -.490, t = 9.665, *p* < .001). Finally, at the vertical collectivism level of +1SD, the effect was the least negative (B = -.383, t = 5.944, *p* < .001, see Fig 3 for visualization). In other words, the higher the vertical collectivism, the less negative the effect of the perceived artificiality of vaccines on people’s vaccination intent. By indicating the above moderating role of vertical collectivism, those results support H1.

### Discussion

In line with the previous research on naturalness bias [2, 44, 45, 46, 48, 49, 51], the results of Study 1 suggest that perceived vaccine artificiality decreases vaccination intent. Crucially, the results indicate the proposed moderating role of vertical collectivism, i.e., diminishing the negative effect of perceived artificiality on vaccination intent. This suggests a potential role of vertical collectivism in combating the negative consequences of perceiving vaccines as artificial. As this perception may be raised by anti-vaxxers’ "unnaturalness" arguments, people low in vertical collectivism may be more prone to those arguments and, thus, may require special attention in the health communication efforts aimed to rebut those arguments.

Study 1 captured an overall belief about vaccine artificiality as a personal characteristic. However, when dealing with a specific vaccine, the artificiality perception may be influenced by the content of marketers’ and policymakers’ communication related to that vaccine, e.g., what features are used to characterize it. Specifically, health communication may emphasize human achievements in intervening in nature with vaccines, and such human-intervention appeals may increase perceived vaccine artificiality, which may, in turn, harm vaccine intent. According to the proposed role of vertical collectivism, we expected that the effect of perceived vaccine artificiality would also be less negative in this setting. Investigating that perspective was the focus of Study 2.

## Study 2

### Method

Study 2 aimed to replicate the test of H1 experimentally. While this hypothesis, predicting that the effect of perceived vaccine artificiality on vaccination intent is less negative for higher levels of vertical collectivism, was supported in Study 1, considering the perceived artificiality of vaccines in general, Study 2 focused on the perceived artificiality of a specific vaccine promoted by vaccine communication. Vaccine marketers and policymakers may emphasize human intervention in nature, referring to the vaccine’s technological advancement, scientific invention, or humankind’s achievement. Those attempts may make people perceive the vaccine as more "artificial" [42, 43, 50, 74]. Therefore, Study 2 took the form of an experiment with perceived vaccine artificiality manipulated by the presence of that human-intervention appeal. Study 2 obtained the required approval from the institutional review board (SGH Warsaw School of Economics Ethical Committee, approval no. 2/2022)

#### Participants

Two hundred three European young adults aged between 18 and 35, working or studying, with at least high school education, and good self-reported English level (M_age_ = 22.591, SD = 2.801, 59.1% females) participated in an online survey (see sample characteristics in S1 Table). Written informed consent was obtained via the online survey form. The researchers had no access to information that could identify individual participants during or after data collection. The participants were recruited by a team of marketing research students, and the sample was convenient and homogenous, like in Study 1. Data were collected in April 2022, when 72.6% of the EU/EEA adult population was vaccinated against COVID-19 with two doses [18]. Additionally, vaccination status (coded as 1 = vaccinated, 0 = unvaccinated) was collected and controlled in the analysis (94.1% of the participants declared vaccinated). The data were accessed for research purposes in May 2022, after the data collection had been completed.

#### Procedure

The design was a single-factor experiment with two conditions of vaccine artificiality (human-intervention appeal present (= high artificiality) vs. absent (= low artificiality)), between-subject. The participants were randomly assigned to the conditions (N_present_ = 111, N_absent_ = 92). As in Study 1, the questionnaire began with the statement that people may be advised to be vaccinated against COVID-19 even if they are already vaccinated. Unlike Study 1, the instruction referred to a particular fictitious COVID-19 vaccine (named "ABC-COVID-VAX") instead of referring to COVID-19 vaccines in general. That was because the human-intervention manipulation took the form of an ad for a particular vaccine, and the aim was to determine the effect of the vaccine description on the response toward the advertised vaccine. It was not expected that one ad would change people’s general views on COVID-19 vaccines. The vaccine name ("ABC-COVID-VAX"), developed within individual discussions with respondents from the study population, was intended to sound possibly neutral without prompting any associations with the constructs involved in the research model, such as artificiality, technology, or collectivistic values.

In the present human-intervention appeal condition (= high artificiality), the participants were exposed to the following description of the advertised vaccine:

"This vaccine consists of unique advanced-technology ingredients triggering specific scientifically-invented processes in your immune system that make your organism significantly more resistant to COVID-19 thanks to the achievements of mankind."

In the above stimuli description, the keywords related to the human-intervention appeal ("advanced-technology ingredients," "scientifically-invented processes in your immune system," and "achievements of mankind") were bolded.

In the absent human-intervention appeal condition (= low artificiality), the stimuli vaccine description differed from the previous condition in that it contained no above human-intervention keywords:

"This vaccine consists of unique ingredients triggering specific processes in your immune system that make your organism significantly more resistant to COVID-19."

Both descriptions stressed the uniqueness of the advertised vaccine. This was intended to ensure the participants would respond to this particular vaccine and not to COVID-19 vaccines in general. To this end, the statement "This vaccine may be dissimilar to other vaccines you know." Appeared on each screen containing the vaccine-related measurements.

After reading the vaccine description, the participants rated their intention to get vaccinated and vaccine artificiality. As the advertised vaccine was fictitious, the instructions asked about "vaccines like ABC-COVID-VAX" (instead of "the ABC-COVID-VAX vaccine") to enhance the realism and easiness of imagining the response situation. Then, vertical collectivism and analytical thinking style were measured. The questionnaire ended with demographics (including COVID-19 vaccination status as an additional control), and the participants were debriefed with the following statement: "Please note that the ABC-COVID-VAX vaccine is a fictitious name. We prepared the advertisement you have seen in this form to understand better how consumers evaluate vaccine ads."

#### Measurements

Vaccination intent was measured as in Study 1, except the statements referred to the advertised vaccine instead of COVID-19 vaccines in general (r = .900, *p* < .001; ρ = .888, *p* < .001). A single item, "COVID-19 vaccines like ABC-COVID-VAX immunize people in a rather artificial way." (similar to Siegrist & Hartmann [91]), coded from 1 = "definitely YES" to 7 = "definitely NO," measured perceived vaccine artificiality (manipulation check). The single-item measurement was applied to focus on an aspect of unnaturalness specifically relevant to the study context (i.e., featuring the technological advancement of and general human intervention related to the vaccine) (cf. [92]). Vertical collectivism was measured like in Study 1 (α = .823), as well as analytical cognitive style (α = .848).

The items included in the measurement scales (vaccination intent, vertical collectivism, and analytical thinking style) were subjected to Confirmatory Factor Analysis (CFA). After dropping two vertical-collectivism items ("Children should be taught to place duty before pleasure." And "Children should feel honored if their parents receive a distinguished award."), the model showed acceptable fit characteristics (χ^2^(48) = 79.828, p < .001, χ^2^/df = 1.565, RMSEA = .053, CFI = 0.974, TLI = 0.967, SRMR = 0.056). In support of the reliability and convergence validity of the measurement scales, Composite Reliabilities (CRs) and Average Extracted Variances (AVEs_ were satisfactory for all scales (CRs ranged from .8 to .9, AVEs ranged from .5 to .9). According to Fornell-Larcker’s criterion, the absolute values of the correlation coefficients between latent variables were lower than the corresponding AVE square roots, and heterotrait-monotrait (HTMT) ratios were below .4 (i.e., within the acceptable threshold of .9), supporting discriminant validity [87]. The factor with the highest eigenvalue in Exploratory Factor Analysis based on the reduced set of indicators (factors extracted using principal component analysis (PCA), Kaiser-Meyer-Olkin (KMO) test for sampling adequacy = .778, Bartlett’s *p* < .001) explained 32.7% of the variance, suggesting no issues with common method bias [88]. The reduced vertical-collectivism scale remained reliable, α = .836 (see details in S5–S7 Tables). For further analysis, each measurement scale was pooled into a single index.

### Results

A moderation analysis was conducted to test H1 (PROCESS model 1 [89], the vaccine artificiality condition was an independent variable (coded as 1 = high artificiality (human-intervention appeal present), 0 = low artificiality (human-intervention appeal absent)), vertical collectivism was a moderator, and vaccination intent served as a dependent variable; analytical thinking style, gender (coded as 1 = female, 0 = male), age, and COVID-19 vaccination status (coded as 1 = vaccinated, 0 = unvaccinated) were covariates (R^2^ = .203, F = 6.160, *p* < .001; variance inflation factors (VIFs) < 1.1). In line with H1, the vaccine artificiality condition × vertical collectivism interaction effect was positive (B = .394, t = 1.950, *p* = .053). The conditional effect of vaccine artificiality condition at the vertical collectivism level of −1SD was negative (B = -.823, t = 2.340, *p* = .020) while being non-significant at the mean and +1SD levels of vertical collectivism. In the same model for the women subsample (R^2^ = .224, F = 4.615, *p* < .001), the above interaction effect was even more visible (B = .556, t = 2.060, *p* = .042; conditional effect of vaccine artificiality condition at the vertical collectivism level of −1SD: B = -1.062, t = 2.148, *p* = .034; conditional effects of vaccine artificiality condition at the mean and +1SD levels of vertical collectivism: NS).

**Fig 4:**
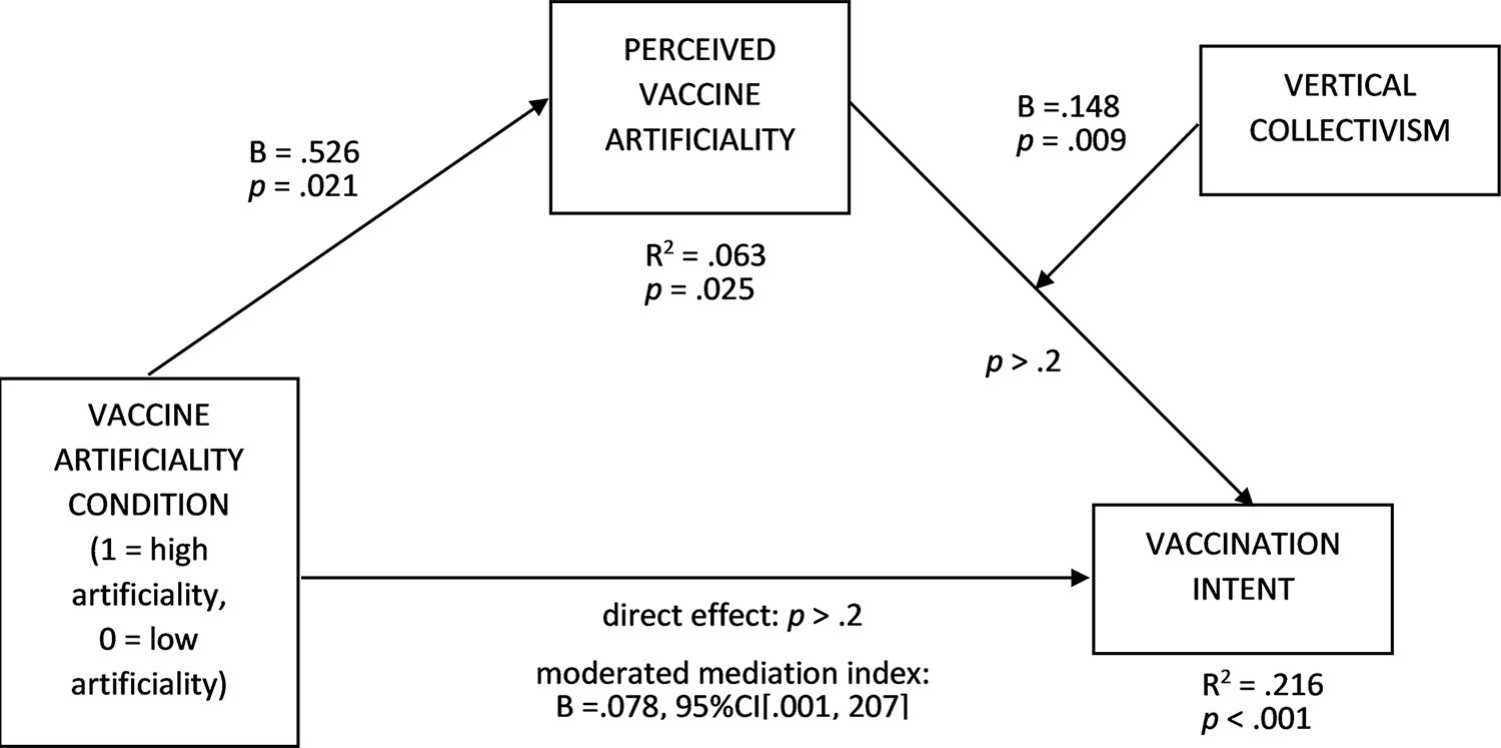
Second-stage moderated mediation effects on vaccination intent (Study 2). Human-intervention appeal was present in the high artificiality condition and absent in the low artificiality condition.

Additionally, a second-stage moderated mediation analysis was conducted to examine better whether the manipulation affected vaccination intent through perceived artificiality (PROCESS model 14 [89], the artificiality condition was an independent variable, perceived vaccine artificiality (manipulation check) was a mediator, vertical collectivism was a moderator, and vaccination intent served as a dependent variable; analytical thinking style, gender, age, and COVID-19 vaccination status were covariates) (Fig 4, R^2^ = .216, F = 6.663, *p* < .001; variance inflation factors (VIFs) < 1.1; 5000 bootstrap samples). The effect of the artificiality condition on perceived vaccine artificiality was positive (B = .526, t = 2.326, *p* = .021), indicating that the manipulation was successful. Moreover, the moderated mediation index was positive (B = .078, 95%CI[.001, .207]). The conditional indirect effects of the artificiality condition on vaccination intent at the vertical collectivism levels of ‒1SD and mean were non-significant while being positive at the +1SD level of vertical collectivism (B = .141, 95%CI[.008, .350]. The direction of moderation is in line H1; however, it turned out that the effect of perceived artificiality, as driven by the human-intervention appeal, may even be positive when vertical collectivism is high.

### Discussion

The results of Study 2 provide further support to the proposition that vertical collectivism diminishes the negative consequences of perceiving vaccines as artificial. While Study 1 demonstrated that the effect of the perceived artificiality of vaccines (in general) is less negative for higher levels of vertical collectivism, Study 2 extended those results dealing with a specific vaccine and the related promotional communication based on human-intervention appeal (i.e., emphasizing the vaccine’s technological advancement and, in general, human intervention behind its development and operation). The results of Study 2 suggest that ‒ in this promotional setting ‒ vertical collectivism may make the effect of perceived vaccine artificiality on vaccination intent even positive. Namely, while the total effect of the human-intervention appeal was significantly negative only for low levels of vertical collectivism, the indirect effect of human-intervention appeal through perceived vaccine artificiality turned out to be significantly positive for high levels of vertical collectivism. This suggests that (1) it is risky to promote a vaccine by emphasizing the vaccine is part of human intervention in nature, as it may increase the perception of the vaccine as artificial, (2) vertical collectivism reduces this harm, making the effect of human-intervention appeal less negative or even more positive, when taking the indirect effect through perceived vaccine artificiality. This way, vertical collectivism may make human-intervention appeal less risky and more persuasive. Therefore, people high in vertical collectivism are more likely to react positively to vaccine communication based on human-intervention appeal.

To sum up, given the current studies, vertical collectivism appears to make people less depreciate vaccine artificiality and improve people’s response to human intervention reference in vaccine communication (i.e., emphasizing technological advances in vaccine development, scientific invention, or humankind’s achievement). In other words, the harmful consequences of perceived vaccine artificiality appear to be shaped socio-culturally by vertical collectivism, which may be a "remedy" for the vaccine "unnaturalness" issue, making people less prone to anti-vaxxers "unnaturalness" arguments and less likely to respond negatively (or more likely to respond positively) to messages promoting a specific vaccine by emphasizing that the vaccine is part of human intervention in nature.

Those results enrich the understanding of the role of collectivism in shaping vaccination intent by revealing that vertical collectivism reduces the negative consequences of perceived vaccine artificiality. Simultaneously, the results offer a better understanding of the perceived role of vaccine artificiality, suggesting that the role is socio-culturally determined by vertical collectivism (see the Theoretical implications section for details). For health communication practitioners, our results suggest that it is worth focusing their vaccine promotional activities on specific socio-culturally defined audiences. Namely, efforts aiming to rebut anti-vaxxers’ "unnaturalness" arguments are likely to be more efficient when targeting people low in vertical collectivism as they tend to be more prone to the negative consequences of perceiving vaccines as artificial. On the other hand, promoting a vaccine by emphasizing human achievements in intervening in nature with the vaccine is likely to be more effective when targeting people high in vertical collectivism, as they tend to react more positively to the perceived artificiality of the promoted vaccine (see the Practical implications section for details).

## Conclusions

### Theoretical implications

The objective of the present research was to examine the possible interplay between perceived vaccine artificiality and vertical collectivism in shaping vaccination intent. Accordingly, the positive interaction effect of perceived vaccine artificiality × vertical collectivism was evidenced in two studies. The first one captured the level of perceiving vaccines, in general, as artificial (as a personal characteristic), and the second one checked the effect of promoting a specific vaccine using human-intervention appeal that increased the vaccine artificiality perception. Our results suggest that, for people higher (vs. lower) in vertical collectivism, (1) the general vaccination intent is less harmed by perceiving vaccines as artificial, and (2) messages promoting a specific vaccine through emphasizing it is part of human intervention in nature (and thus, accentuating the vaccine’s artificiality) are more persuasive. In sum, our research indicates that vertical collectivism reduces the negative effect of perceived vaccine artificiality on vaccination intent and, in the case of promoting vaccines through human-intervention appeal, makes the effect of perceived vaccine artificiality on vaccination intent even positive. This way, the current research adds to the growing literature on perceived naturalness/artificiality [2, 40, 41, 44–46, 48–49, 51] by discerning the moderating role of vertical collectivism in the effect of perceived vaccine artificiality (i.e., showing that vaccine naturalness bias may be reduced by vertical collectivism). As such, we introduce another worldview moderator of naturalness bias, compared to the moderating role of natural connectedness and Taoism evidenced for drugs by Cao & Li [93] and Li & Cao [94]. By proposing and evidencing a link between collectivism, perceived vaccine artificiality, and vaccination intent, the current research contributes to the vaccination behavior literature related to the role of collectivism in vaccination attitudes and intentions [4–12] and, more generally, to the recent research on social factors of COVID-19 vaccination intent (e.g., [95, 96]). Our research adds to the extant health communication literature related to vaccines (e.g., [24, 25, 27–34]) by demonstrating the effects of vaccine human-intevention appeal considering the mediating role of perceived vaccine artificiality and a moderating role of vertical collectivism. Finally, showing the role of vertical collectivism in the consequences of vaccine artificiality perception, we contribute to the cultural dimension literature related to collectivism (e.g., [16, 53, 54]).

### Practical implications

For health communication, the current research has twofold implications. First, it suggests that people with lower vertical collectivism are potentially more prone to anti-vaccine arguments based on "unnaturalness," as their increased level of perceived vaccine artificiality is more likely to decrease vaccination intent. Therefore, the efforts of vaccine policymakers and marketers to refute those "unnaturalness" arguments by emphasizing that vaccines contain natural components and operate through natural processes of immunization [97] should be targeted primarily at people with low vertical collectivism. Identifying those people may be supported by social media data. For example, user collectivism was demonstrated to be positively associated with the use of artistic hashtag styles on social media platforms [98]. Another attempt to make people less prone to "unnaturalness" arguments may be activating their collectivistic values with simple stimuli embedded in communication [5, 6]. Specifically, endorsing social hierarchy (e.g., respect for high-level social groups) in vaccination promotion could make the issue of vaccine "unnaturalness" less important to people, providing a better ground to challenging the supremacy of "natural" immunity over the "artificial" one, as proposed by World Health Organization (WHO) health communication guidelines [99].

Second, the current results indicate that for people with higher vertical collectivism, the increased level of perceived vaccine artificiality, which can be caused by human-intervention appeal (e.g., emphasizing an advanced technology standing behind vaccines), is more likely to enhance vaccination intent. Therefore, vaccine communication based on such appeal should be focused on people with high vertical collectivism or supported by collectivistic values activation.

In sum, health communication referring to vaccine artificiality (e.g., rebutting vaccine "unnaturalness" arguments or emphasizing human intervention, e.g., in the form of advanced technologies) should consider different levels of vertical collectivism within audiences.

### Limitations and further research directions

A collectivistic mindset may be situationally determined and experimentally manipulated [53], which was also applied to the vaccination context [5, 6]. However, the current research did not manipulate collectivism. Further studies on the role of perceived vaccine artificiality in vaccine attitudes can involve such manipulation. It may strengthen the causal evidence of the proposed moderating role of collectivism and the effectiveness of pro-vaccine campaigns referring to collectivism. Such experimental results could provide actionable guidelines for vaccine communication, where rebutting "unnaturalness" anti-vaxxers’ arguments or human-intervention appeal is accompanied by activating vertical collectivist values to enhance effectiveness.

The current research did not manipulate the exposition on "unnaturalness" arguments against vaccines. Future studies can apply such manipulation to investigate whether, in line with the moderating role of collectivism proposed here, the "unnaturalness" arguments influence vaccination intent more negatively among people with low vertical collectivism.

Both studies were based on contrived scenarios of a hypothetical need to be vaccinated against COVID-19. Therefore, further studies on the topic may attempt to improve external validity by dealing with natural settings, e.g., in the form of field experiments with different types of messages about a specific vaccine (e.g., disseminated through social media), measuring the actual outcomes in the form of interest in or intake of the vaccine.

Future research should investigate the moderating role of collectivism in the effects of vaccine artificiality perception across various geographical regions (e.g., differing in cultural collectivism) and demographic characteristics (e.g., including elderly people). The proposed conceptual model should be examined in the endemic stage of the COVID-19 pandemic and for other preventable illnesses.

Our research focused on one type of behavioral outcome, i.e., vaccination intent. However, other forms of vaccine patronage are important in the vaccination context. For example, vaccine advocacy [100] is desirable as positive word-of-mouth on vaccinations may support vaccine promotional efforts. Thus, future studies should examine whether vertical collectivism reduces the effects of perceived vaccine artificiality on vaccine advocacy.

Finally, our theorization may suggest that vertical collectivism reduces the negative effect of perceived artificiality for other interventions that may be perceived as societal efforts made by high-level social groups (such as scientists, engineers, or other experts and institutions) as means to combat health problems (e.g., drugs, preventative tests, or procedures like in-vitro fertilization) and social/environmental issues (like cultured meat), or to develop human civilization (e.g., AI-based technological solutions). Examining those generalizations should be considered a promising path for further research efforts.

## Supporting information

S1 Table Sample characteristics (Study 1 and Study 2). (DOCX)

S2 Table Confirmatory factor analysis for the measurement scales in Study 1. (DOCX)

S3 Table Fornell-Larcker’s criterion: Correlations between the latent variables in Study 1. Bolded figures represent AVE square roots.
(DOCX)

S4 Table Heterotrait-monotrait (HTMT) ratios for latent variables in Study 1. (DOCX)

S5 Table Confirmatory factor analysis for the measurement scales in Study 2. (DOCX)

S6 Table Fornell-Larcker’s criterion: Correlations between the latent variables in Study 2. Bolded figures represent AVE square roots.
(DOCX)

S7 Table Heterotrait-monotrait (HTMT) ratios for latent variables in Study 2. (DOCX)
