# Correction: How does collectivism help deal with perceived vaccine artificiality? The case of COVID-19 vaccination intent in European young adults

**DOI:** 10.1371/journal.pone.0304372

**Published:** 2024-05-22

**Authors:** Wojciech Trzebiński, Jerzy Trzebiński

The ORCID iD is missing for the second author. Please see the author’s ORCID iD here:

- Author Jerzy Trzebiński’s ORCID iD is: 0000-0003-3948-1676 (https://orcid.org/0000-0003-3948-1676).

There are errors in the funding statement. The correct funding statement is as follows: This study was supported by the Polish Ministry of Education and Science (grant no. KZiF/S22:1.58), SGH Warsaw School of Economics, Collegium of Management and Finance. The open access publication was funded by SWPS University Research Development Funds. The funders had no role in the study design, data collection and analysis, decision to publish, or preparation of the manuscript.

## References

[1] Trzebiński W, Trzebiński J (2024) How does collectivism help deal with perceived vaccine artificiality? The case of COVID-19 vaccination intent in European young adults. PLoS ONE 19(3): e0300814. 10.1371/journal.pone.0300814. 38502651 PMC10950243

