# Supplementary material for: How does collectivism help deal with perceived vaccine artificiality? The case of COVID-19 vaccination intent in European young adults
Source: PLoS One. 2024 Mar 19;19(3):e0300814. doi: 10.1371/journal.pone.0300814 (PMC10950243; doi:10.1371/journal.pone.0300814)
Supplement: S1 Table — (DOCX) [file pone.0300814.s001.docx]

S1 Table. Sample characteristics (Study 1 and Study 2).

|  | **Study 1** | | **Study 2** | |
| --- | --- | --- | --- | --- |
|  | Frequency | Percent | Frequency | Percent |
| **Gender** | | | | |
| **Females** | 225 | 53.8 | 120 | 59.1 |
| **Males** | 193 | 46.2 | 83 | 40.9 |
| **Age** | | | | |
| **<20** | 25 | 6.0 | 15 | 7.4 |
| **20-24** | 336 | 80.4 | 152 | 74.9 |
| **25-29** | 40 | 9.6 | 29 | 14.3 |
| **≥30** | 17 | 4.1 | 7 | 3.4 |
| **Education (the highest level completed)** | | | | |
| **High school** | 231 | 55.3 | 102 | 50.2 |
| **University** | 187 | 44.7 | 101 | 49.8 |
| **Occupation** | | | | |
| **Working** | 140 | 33.5 | 77 | 37.9 |
| **Studying** | 351 | 84.0 | 165 | 81.3 |
