# Supplementary material for: How does collectivism help deal with perceived vaccine artificiality? The case of COVID-19 vaccination intent in European young adults
Source: PLoS One. 2024 Mar 19;19(3):e0300814. doi: 10.1371/journal.pone.0300814 (PMC10950243; doi:10.1371/journal.pone.0300814)
Supplement: S2 Table — (DOCX) [file pone.0300814.s002.docx]

S2 Table. Confirmatory Factor Analysis for the measurement scales in Study 1.

| **Latent variable** | **Measurement item** | **Factor loading** | **Cronbach’s Alpha** | **CR** | **AVE** |
| --- | --- | --- | --- | --- | --- |
| **Vaccination intent** | I intend to get vaccinated for COVID-19. | .973 | r = .8 ρ = .8 | .9 | .8 |
|  | If the COVID-19 vaccine is free, I like to get the vaccine. | .865 |  |  |  |
| **Perceived vaccine artificiality** | Although human-created, COVID-19 vaccines immunize people in a rather natural way. (reverse-coded) | .705 | r = .5 ρ = .5 | .7 | .5 |
|  | COVID-19 vaccines use natural components and mechanisms. (reverse-coded) | .728 |  |  |  |
| **Vertical collectivism** | I would sacrifice an activity that I enjoy very much if my family did not approve it. | .850 | α = .8^1^ | .8 | .5 |
|  | I would do what would please my family, even if I disliked that activity. | .799 |  |  |  |
|  | I usually sacrifice my self-interest for the benefit of my group. | .581 |  |  |  |
|  | We should keep our aging parents with us at home. | .525 |  |  |  |
| **Analytical thinking style** | I try to use as much information on pros and cons as possible. | .793 | α = .8 | .8 | .6 |
|  | I carefully compare the options I have on several different aspects. | .883 |  |  |  |
|  | I rely on facts rather than on general impressions and feelings. | .585 |  |  |  |
|  | My decision is based on careful thinking and reasoning. | .750 |  |  |  |

^1^ The Cronbach alpha value refers to the final scale after dropping the items with the lowest factor loadings.
