# Supplementary material for: How does collectivism help deal with perceived vaccine artificiality? The case of COVID-19 vaccination intent in European young adults
Source: PLoS One. 2024 Mar 19;19(3):e0300814. doi: 10.1371/journal.pone.0300814 (PMC10950243; doi:10.1371/journal.pone.0300814)
Supplement: S4 Table — (DOCX) [file pone.0300814.s004.docx]

S4 Table. Heterotrait-monotrait (HTMT) ratios for latent variables in Study 1.

| **Latent variable** | **Vaccination intent** | **PVA** | **VC** |
| --- | --- | --- | --- |
| **Perceived vaccine artificiality (PVA)** | -.594 |  |  |
| **Vertical collectivism (VC)** | .156 | -.213 |  |
| **Analytical thinking style** | .286 | -.319 | .146 |
