# Supplementary material for: How does collectivism help deal with perceived vaccine artificiality? The case of COVID-19 vaccination intent in European young adults
Source: PLoS One. 2024 Mar 19;19(3):e0300814. doi: 10.1371/journal.pone.0300814 (PMC10950243; doi:10.1371/journal.pone.0300814)
Supplement: S5 Table — (DOCX) [file pone.0300814.s005.docx]

S5 Table. Confirmatory Factor Analysis for the measurement scales in Study 2.

| **Latent variable** | **Measurement item** | **Factor loading** | **Cronbach’s Alpha** | **CR** | **AVE** |
| --- | --- | --- | --- | --- | --- |
| **Vaccination intent** | I intend to get vaccinated for COVID-19. | .950 | r = .9 ρ = .9 | .9 | .9 |
|  | If the COVID-19 vaccine is free, I like to get the vaccine. | .947 |  |  |  |
| **Vertical collectivism** | I would sacrifice an activity that I enjoy very much if my family did not approve it. | .819 | α = .8^1^ | .8 | .5 |
|  | I would do what would please my family, even if I disliked that activity. | .788 |  |  |  |
|  | Before taking a major trip, I consult with most members of my family and many friends. | .562 |  |  |  |
|  | I usually sacrifice my self-interest for the benefit of my group. | .733 |  |  |  |
|  | I hate to disagree with others in my group. | .553 |  |  |  |
|  | We should keep our aging parents with us at home. | .615 |  |  |  |
| **Analytical thinking style** | I try to use as much information on pros and cons as possible. | .769 | α = .8 | .8 | .6 |
|  | I carefully compare the options I have on several different aspects. | .786 |  |  |  |
|  | I rely on facts rather than on general impressions and feelings. | .704 |  |  |  |
|  | My decision is based on careful thinking and reasoning. | .800 |  |  |  |

^1^ The Cronbach alpha value refers to the final scale after dropping the items with the lowest factor loadings.
