# Supplementary material for: How does collectivism help deal with perceived vaccine artificiality? The case of COVID-19 vaccination intent in European young adults
Source: PLoS One. 2024 Mar 19;19(3):e0300814. doi: 10.1371/journal.pone.0300814 (PMC10950243; doi:10.1371/journal.pone.0300814)
Supplement: S6 Table — Bolded figures represent AVE square roots. (DOCX) [file pone.0300814.s006.docx]

S6 Table. Fornell-Larcker’s criterion: correlations between the latent variables in Study 2. Bolded figures represent AVE square roots.

| **Latent variable** | **VI** | **VC** | **AN** |
| --- | --- | --- | --- |
| **Vaccination intent (VI)** | **.948** |  |  |
| **Vertical collectivism (VC)** | .304 | **.687** |  |
| **Analytical thinking style (AN)** | .073 | .189 | **.765** |
