# Supplementary material for: How does collectivism help deal with perceived vaccine artificiality? The case of COVID-19 vaccination intent in European young adults
Source: PLoS One. 2024 Mar 19;19(3):e0300814. doi: 10.1371/journal.pone.0300814 (PMC10950243; doi:10.1371/journal.pone.0300814)
Supplement: S7 Table — (DOCX) [file pone.0300814.s007.docx]

S7 Table. Heterotrait-monotrait (HTMT) ratios for latent variables in Study 2.

| **Latent variable** | **Vaccination intent** | **VC** |
| --- | --- | --- |
| **Vertical collectivism (VC)** | .339 |  |
| **Analytical thinking style** | .074 | .225 |
